# FAIR data representation in times of eScience: a comparison of instance-based and class-based semantic representations of empirical data using phenotype descriptions as example

**DOI:** 10.1186/s13326-021-00254-0

**Published:** 2021-11-25

**Authors:** Lars Vogt

**Affiliations:** TIB Leibniz Information Centre for Science and Technology, Welfengarten 1B, 30167 Hanover, Germany

**Keywords:** Phenotype data, Phenotype knowledge graph, Semantic phenotype, Ontology, Knowledge management, Semantic graph, Data representation, FAIR data, ABox expression, TBox expression

## Abstract

**Background:**

The size, velocity, and heterogeneity of Big Data outclasses conventional data management tools and requires data and metadata to be fully machine-actionable (i.e., eScience-compliant) and thus findable, accessible, interoperable, and reusable (FAIR). This can be achieved by using ontologies and through representing them as semantic graphs. Here, we discuss two different semantic graph approaches of representing empirical data and metadata in a knowledge graph, with phenotype descriptions as an example. Almost all phenotype descriptions are still being published as unstructured natural language texts, with far-reaching consequences for their FAIRness, substantially impeding their overall usability within the life sciences. However, with an increasing amount of anatomy ontologies becoming available and semantic applications emerging, a solution to this problem becomes available. Researchers are starting to document and communicate phenotype descriptions through the Web in the form of highly formalized and structured semantic graphs that use ontology terms and Uniform Resource Identifiers (URIs) to circumvent the problems connected with unstructured texts.

**Results:**

Using phenotype descriptions as an example, we compare and evaluate two basic representations of empirical data and their accompanying metadata in the form of semantic graphs: the class-based TBox semantic graph approach called Semantic Phenotype and the instance-based ABox semantic graph approach called Phenotype Knowledge Graph. Their main difference is that only the ABox approach allows for identifying every individual part and property mentioned in the description in a knowledge graph. This technical difference results in substantial practical consequences that significantly affect the overall usability of empirical data. The consequences affect findability, accessibility, and explorability of empirical data as well as their comparability, expandability, universal usability and reusability, and overall machine-actionability. Moreover, TBox semantic graphs often require querying under entailment regimes, which is computationally more complex.

**Conclusions:**

We conclude that, from a conceptual point of view, the advantages of the instance-based ABox semantic graph approach outweigh its shortcomings and outweigh the advantages of the class-based TBox semantic graph approach. Therefore, we recommend the instance-based ABox approach as a FAIR approach for documenting and communicating empirical data and metadata in a knowledge graph.

## Background

More than 90% of today’s data have been created within the past two years, with more than 2.5 million new scientific papers being published each year [1–4]. High-throughput technologies, social media, mobile devices, digital imaging, sensors, and the Internet of Things, all contribute to Big Data in science and everyday life, allowing researchers to answer questions that could not be answered before. This new driving force for scientific progress in data-rich fields of empirical research has been called **data exploration** or **eScience** [5].

In times of pressing societal, technological, economic, and ecological challenges that arise from climate change, diversity loss, and the COVID-19 outbreak, the need for continuously monitoring key parameters and rapidly analyzing large amounts of empirical and thus observation- and measurement-based data and metadata has become clear. In most of the cases, this requires first **integrating** datasets from various sources and from diverse research communities before they can be analyzed. In general, data management tools and with them data and metadata formats and standards have become increasingly important to support various eScience workflows.

Whereas Big Data brings to us new opportunities for research, it also comes with new challenges that arise from the change in size, velocity, and variety of data, outclassing the capabilities of conventional methods and techniques of data management and analysis [6]. To be most efficiently usable, data and metadata therefore must be maximally **F**indable, **A**ccessible, **I**nteroperable, and **R**eusable and thus comply with the **FAIR Guiding Principles** [7]. A central aspect of making data and metadata FAIR and fully eScience-compliant is making them machine-actionable through using Semantic Web^1^ technologies such as ontologies [8, 9]. Ontologies and other controlled vocabularies are important because they provide a framework for integrating and documenting data and metadata in the standardized semantic structure that eScience and the FAIR Guiding Principles require [10].

**Ontologies** are dictionaries that are used for describing a certain reality. They consist of terms with commonly accepted definitions that are formulated in a highly formalized canonical syntax and standardized format, such as the Web Ontology Language^2^ (OWL) serialized to the Resource Description Framework^3^ (RDF), with the goal to yield a lexical or taxonomic framework for knowledge representation [11]. OWL is based on description logics (DL), which provides a logical formalism for ontologies. DL distinguishes **TBox** expressions that contain universal statements about classes and **ABox** expressions that contain assertions about instances^4^ [12]. Both ABox and TBox expressions can be represented as semantic graphs using RDF’s triple syntax of *Subject*, *Predicate*, and *Object*. A **semantic graph** is a network of RDF/OWL-based triple statements, in which a given Web resource can take the *Object* position in one triple and the *Subject* position in another triple, thereby connecting the triples to form a connected graph.

When we understand ontologies as modeling commonly accepted domain knowledge about specific *kinds* of entities and their properties and relations, expressed as classes and class axioms^5^ that are defined through universal statements^6^ [13, 14], ontologies consist of TBox expressions and not ABox expressions and thus do not contain statements about *individual entities*. Ontologies in this sense, therefore, do not contain actual empirical data. But one can employ ontology terms in ABox expressions, for instance for stating that a given individual entity is of a particular kind and that it therefore represents an instance of the respective ontology class.

Each ontology class, individual entity, and property possesses its own unique and persistent Uniform Resource Identifier^7^ (URI), through which it can be identified and individually referenced in various contexts. By providing URIs and machine-readable definitions for their classes, ontologies can be used to substantially increase semantic transparency and machine-actionability for all kinds of information, including empirical data. The URIs of ontology classes are often used for semantically enriching documents for data mining purposes of historical literature and for annotating database contents to improve integration and interoperability of data and thus computability of contemporary empirical data.

Using Semantic Web technologies, ontologies can be employed to express, document, and represent empirical data as structured, interlinked, and semantically rich semantic graphs that substantially improve the findability, accessibility, interoperability, and reusability of data, thus making data compliant with the FAIR Guiding Principles [7]. This is becoming increasingly important in the age of Big Data and Linked Open Data and allows data and metadata to be used in eScience [8, 9, 15–19]. Ontologies are thus cornerstones of the Semantic Web and provide solutions to various problems of information and knowledge management, including word-sense disambiguation, standardization, and measuring semantic similarity, providing an efficient framework for question answering, knowledge representation, natural language processing, and semantic searches [20–28].

### Real particulars, real universals, and their textual representations

Documenting empirical data in the form of semantic graphs attempts to represent a particular portion of reality and is, as such, a semiotic process. When reflecting on the way we do research and especially when comparing different approaches of representing empirical reality, it is good to distinguish basic categories of entities that are involved in this semiotic process [9, 29–31].

Empirical data attempt to represent real entities and their relations. **Real entities** are material objects, processes, qualities, and states that exist in reality, independent of any human mind. Any real entity is either a **particular** (i.e., instance, individual, token) or a **universal** (i.e., kind, type). Particulars—e.g. the planet *Earth* or *you*, the reader, and *I*—are singly located entities that are bound to a specific location in space and time, whereas universals—e.g. *cell* or *multicellular organism*—are multiply located entities that exist in their corresponding particulars [32, 33]. A universal is thus anything that is instantiated by particulars and a particular anything that instantiates a universal [34]. In this sense, *I* am an instance of *multicellular organism*.

Real entities do not exist inside of our minds but outside in the real world. When we think of a real entity, we generate a **cognitive representation** referring to it in the form of thoughts, perceptions, concepts, ideas, and beliefs. When we communicate information about a real entity with somebody else, we want that person to share a maximally similar cognitive representation about the entity. In doing so, we often use language and thus terms and statements for describing our cognitive representations.

Any term and statement referring to a real entity is a **textual representation of a real entity** and must be distinguished not only from the real entity it refers to but also from the cognitive representation it should induce. Based on the distinction of the two basic categories of real entities, i.e., particulars and universals, we can distinguish between textual representations of particulars in the form of proper names and assertional statements and textual representations of universals in the form of kind terms (also called general terms) and universal statements. **Proper names** refer to particulars and usually have no textual definitions but only assertional statements associated with them. **Assertional statements** are statements that claim to be only true for a specific particular. If assertional statements are grounded in empirical knowledge that is based on observation and experimentation, we refer to them as **empirical data**. Empirical data can be formulated in OWL and documented in the form of **instance-based ABox semantic graphs**, in which particular real entities can be referred to through assigning them their own URIs, and their class affiliation can be specified by referencing the URI of the respective ontology class.

A **kind term**, on the other hand, is usually associated with a concept in the form of a **class** that defines the meaning of the term by means of universal statements. A **universal statement** represents commonly accepted **domain knowledge** and claims to be true for all instances of the kind the statement is referring to. Scientific theories, but also definitions of ontology classes using axioms [35], are examples of universal statements. Ontologies contain universal statements that can be formulated in OWL and documented in the form of **class-based TBox semantic graphs**.

These two types of textual representations can for instance be applied to describing anatomical phenotypes. The resulting descriptions attempt to represent the organization of real anatomical entities (cf. anatomical entity^8^ of *Uber Anatomy Ontology*; id UBERON:0001062) in the form of textual representations. Each description consists of at least one descriptive statement. We here understand a **descriptive statement** as the **smallest semantically meaningful unit of empirical information**. Descriptive statements can be differentiated based on their semantic content into assertional and universal statements [13, 14], e.g. the description of the essential properties^9^ of a compound eye as a set of universal statements defining the class compound eye or the description of an individual compound eye possessing a particular set of properties documented in a set of assertional statements.

For reasons of efficiency and simplicity, descriptions of particulars usually always involve references to ontology classes. Stating that a given particular entity is an instance of the class compound eye, for example, implies that all defining properties of the class ‘compound eye’ also necessarily apply to this particular entity. To which degree information is provided through class affiliations thereby depends on several factors, including the anatomical variability of the Operational Descriptive Unit (ODU),^10^ the frame of reference of the description and which relevant ontology terms are available. However, since an instance of a class necessarily has all the class-defining properties, the reference to ontology classes within a description logically and semantically represents an implicit short form for what can be explicitly expressed in an instance-based ABox semantic graph. Regardless, empirical data necessarily and always have to include some ABox expression, even if this may only be a statement about some individual entity instantiating some ontology class.

Here, we discuss and evaluate the conceptual differences between two approaches of semantically representing empirical data using semantic graphs, i.e., an instance-based approach that represents data in the form of ABox expressions and a class-based approach that represents them in the form of TBox expressions. We use phenotype descriptions as an example for this comparison.

### Phenotypes, canonical anatomy, instance anatomy, and the use of ontologies for documenting phenotype descriptions

The Phenotype of an organism refers to its observable constituents, properties, and relations that can be considered to result from the interaction of the organism’s genotype with itself and its environment. Anatomy is the part of the phenotype that refers to the physical and structural properties of the organism. Anatomical data are the primary source of evidence for defining most species, for understanding their phylogeny, for recognizing, defining, and diagnosing pathological conditions in plants, animals, and other organisms, and they provide valuable insights into the development, function, evolution, and interaction of phenotypes with their environments [36, 37].

In anatomy, we distinguish canonical anatomy and instantiated anatomy. **Canonical anatomy** is *“a field of anatomy (science) that comprises the synthesis of generalizations based on anatomical observations that describe idealized anatomy (structure)”*, whereas **instance anatomy** is *“the field of anatomy (science) which comprises anatomical data pertaining to instances (i.e., individuals) of organisms and their parts”* ( [38] p. 480; see also, e.g., [39, 40]). While *instance anatomy* aims at representing the actual anatomical organization of a particular organism or a particular anatomical entity as it can be observed, resulting in what could be called ‘factual’ descriptions [9, 31], *canonical anatomy* aims at representing the typical anatomical organization of the members of a certain taxon or typical exemplar instances of a specific kind of anatomical entity. Canonical anatomy is applied in contexts in which deviation from a defined ‘normal’ condition is important, for instance, in medical contexts or when studying mutants against a canonical wild-type [41–45].

Information typically belonging to canonical anatomy is commonly accepted domain knowledge in the form of universal statements about kinds of anatomical entities that can be found in ontologies and can be represented as class-based TBox semantic graphs (see also *invariants* in [46]), whereas information typically belonging to instance anatomy is empirical anatomical data in the form of assertional statements about particular anatomical entities that can be found in phenotype descriptions of individual specimens and can be represented as instance-based ABox semantic graphs.

Unfortunately, despite their importance to life sciences and beyond, anatomical data are usually still published as anatomical descriptions using natural language and thus in the form of unstructured texts. The descriptions are not machine-actionable and often hidden behind pay-walls. This substantially impedes the findability and accessibility of anatomical data. Moreover, due to the immanent semantic ambiguity of anatomical terminology, researchers not familiar with the described taxon and its associated anatomical literature will have substantial problems comprehending and interpreting anatomical data [47]. The meaning of terms is often taxon-, author-, and time-dependent. And while some terms refer to a set of common spatio-structural properties, others refer to a common function, a common developmental pathway, or a presumed common evolutionary origin, or some mixture of these. The same applies to phenotype descriptions in general. The semantic ambiguity of phenotype descriptions that are based on natural language substantially limits the interoperability and reusability of phenotype data, with the consequence that phenotype data usually do not comply with the FAIR guiding principles.

It has been demonstrated that phenotype descriptions can be represented using ontology terms with RDF’s triple syntax of *Subject*, *Predicate*, and *Object* and stored as semantic graphs [47–56]. Two alternative basic approaches have been employed for representing the anatomical organization of a given specimen using ontology terms: a class-based TBox and an instance-based ABox approach. The applicability of these two approaches is not limited to phenotypic data but can be used for representing any type of empirical data. The two approaches differ mainly in technical details that have substantial practical consequences in terms of their respective applicability and can also be aligned to underlying conceptual differences resulting from different research contexts.

**Fig. 1:**
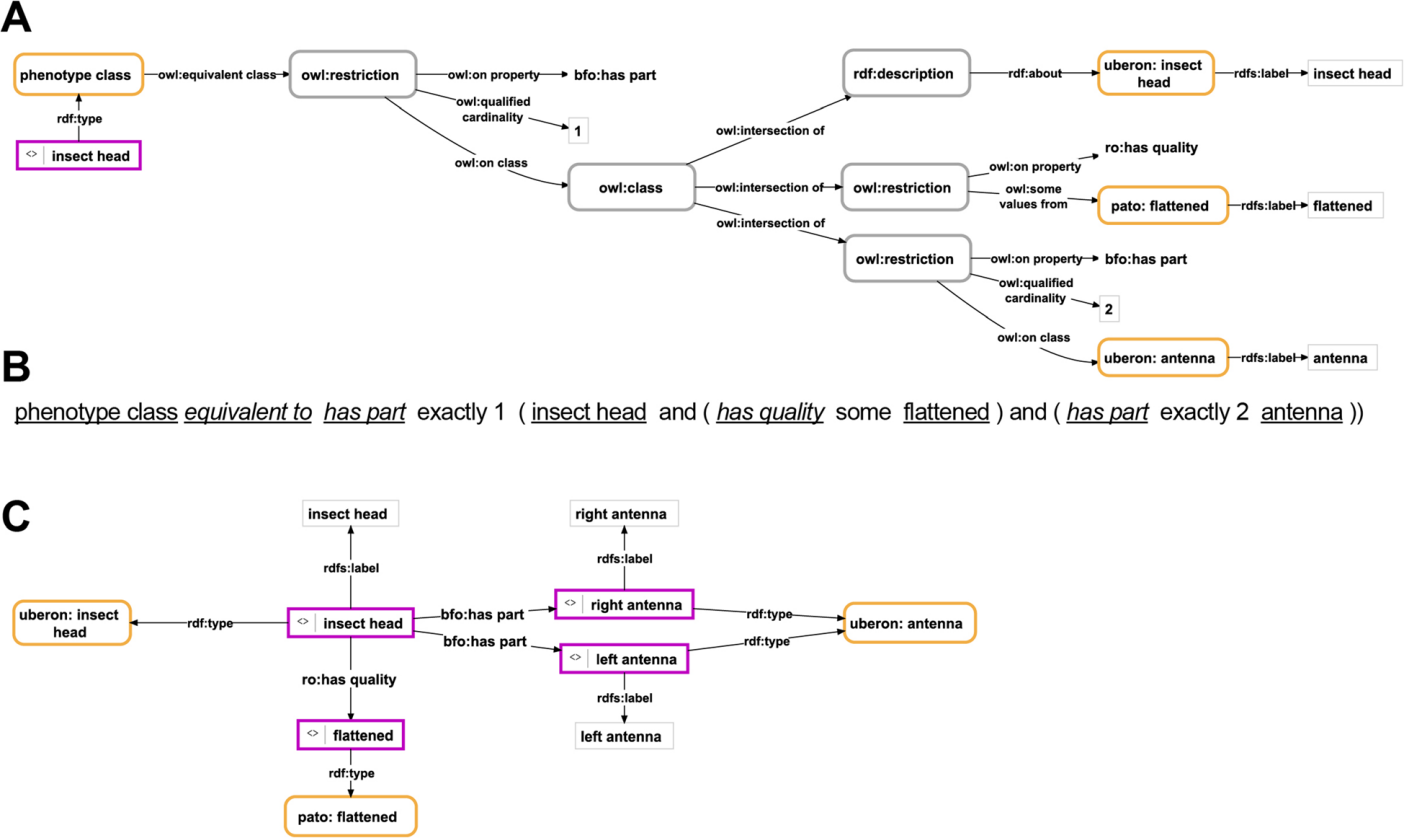
Phenotype description of an insect head with a flattened shape and with two antennae in the form of a class-based TBox semantic graph, OWL Manchester Syntax, and an instance-based ABox semantic graph. **A**: The class-based TBox semantic graph description of an insect head with a flattened shape that has two antennae as its parts. It consists of an instance (purple-bordered box) that instantiates the phenotype class that contains the actual description of the phenotype (yellow-bordered box) in the form of a class axiom consisting of anonymous property restrictions and class descriptions (grey-bordered boxes). The class axiom characterizes all instances of the class to consist of exactly one instance of insect head (id UBERON:6000004) that has the quality of a flattened (id PATO:0002254) shape and that has as its parts exactly two instances of antenna (id UBERON:0000972). **B**: An alternative format representing the class axioms from the ‘phenotype class’ from (A) expressed in OWL Manchester Syntax [60] (ontology classes shown with their label underlined, ontology properties with their label in italics and underlined, ‘and’ being used in the sense of intersection of two mathematical sets and ‘exactly’ as a cardinality specification). **C**: The instance-based ABox semantic graph description of an insect head with a flattened shape that has two antennae as its parts. It consists of the instance of the class insect head (id UBERON:6000004), the instance of the class flattened (id PATO:0002254), which relates to the head as its quality, and two instances of the class antenna (id UBERON:0000972), which relate to the head as its parts. Labels (in light-grey-bordered boxes) indicate how the different instances should be represented in a human-readable format. For reasons of clarity, resources are not represented with their URIs but with their human-readable labels. *Purple-bordered box = instance resource; yellow-bordered box with rounded corners = ontology class resource; grey-bordered box with rounded corners = anonymous class; light-grey-bordered box = literal or numerical value; labeled arrow = property resource*

In the **class-based TBox approach**, a specific anatomical phenotype is described in reference to a specific ontology class, which in turn is defined according to the set of properties that are characteristic to the respective phenotype (Fig. 1). The definition of the class takes the form of an Entity–Quality (EQ) expression and provides the description for that particular phenotype in the form of a set of TBox expressions. Respective descriptions have been called **Semantic Phenotypes**, in which the ODU is specified as instantiating a specific phenotype ontology class [48, 50–52].

In the **instance-based ABox approach**, the anatomical phenotype is not described within the definition of a single ontology class, but instead in the form of a detailed semantic graph, built from ABox expressions that consist of several instance resources (i.e., URIs), each of which refers to a particular part or property/quality of the ODU (Fig. 1C). The resources themselves thus represent instances and not classes, but they instantiate ontology classes. We term the resulting descriptions **Phenotype Knowledge Graphs**, and they follow a more modular framework that makes use of anatomical entity terms and property/quality terms from existing ontologies [29, 47, 49, 57–59].

In the following, we start by specifying the requirements that empirical data must satisfy in the age of eScience and Big Data, with phenotype data as an example. Based on these distinctions and requirements, we introduce and compare the class-based and the instance-based approach for documenting empirical data in the form of semantic graphs. We provide some historical background on how the class-based approach evolved to explain why certain conceptual choices have been made. We discuss the contexts in which the two approaches can be applied and discuss and evaluate the technical differences between them and their practical consequences within the field of anatomy. We think that with their considerable complexity and heterogeneity, covering quantitative (measurements) as well as qualitative (form, shape) information, including contextual information in the form of descriptions of a specimen relative to some other specimen, phenotype descriptions provide a well-suited framework for comparing and evaluating the benefits and shortcomings of instance-based and class-based semantic graphs as two basic approaches of semantically representing empirical data. Finally, we discuss the conceptual suitability of both approaches regarding meeting eScience-compliant standards and the FAIR Guiding Principles.

We want to emphasize that we do not intend to compare the overall benefits and problems of representing and using data or knowledge in the form of ABox and TBox expressions in general. We rather focus in our comparison on the context of documenting and managing empirical data and thus the results from observations, measurements, and experimentation.

## Methods

In the age of eScience and Big Data, empirical data must meet certain technical requirements to be able to take full advantage of the benefits that semantic analytical frameworks offer. They should be **easily findable**, **accessible**, and **explorable** for human readers in respective online data repositories and actionable for machines alike. In case of phenotype data, a researcher should be able to query a phenotype repository using detailed searches, e.g., for descriptions of heads that possess a specific type of antenna and that have a weight larger than 10 mg, restrict this search to a specific taxonomic group and retrieve a list of corresponding descriptions, preferably associated with images supporting the findings. Ideally, we need something comparable to a **BLAST search for phenotypes** that enables finding descriptions in a repository that are maximally similar to an input description.

Not only empirical data themselves should be findable, accessible, and explorable but also all **relevant associated metadata**, amongst others to be able to evaluate the **trustworthiness** and **credibility** of the data.

Non-experts should be able to understand and interpret empirical data correctly, just like researchers can do today with DNA sequence data without having to be a molecular biologist by profession. Data representations thus must be **semantically transparent** in the sense that they make the meaning of terms used in the representation readily available.

Data representations should also be **comparable,** and it should be possible to **expand** them and **complement** them with additional and more detailed information. Moreover, because humans make mistakes, we need an effective way in which researchers can **correct mistakes** in data representations and thereby transparently track what has been changed.

It should be possible to **integrate different frames of reference** (in the case of anatomical data, possible frames of reference would be, i.e., structural, functional, developmental, and evolutionary anatomy) within descriptive empirical data and to **fragment a description** into smaller parts to reuse only those parts of the description that are of interest for a given context.

If we establish a general, domain-specific standard for representing descriptive empirical data, this standard should be **universally usable** and **reusable**, i.e., the syntax, format, and other standards associated with descriptive data should not be specifically tailored for a particular analytical framework such as phylogenetics within the domain of anatomy. Researchers should be able to use descriptive data in the scientific context that is relevant to them.

Last, but not least, researchers should also be able to generate empirical data in the most time-efficient way. If for instance phenotype descriptions themselves would be completely machine-actionable, we could develop tools and algorithms that facilitate the **semi-automatic generation of phenotype descriptions from images**, therewith widening one of the most problematic bottlenecks in anatomy: generating anatomical phenotype descriptions and thus anatomical data.^11^ If descriptions are machine-actionable, we can also start to **parameterize** the **analysis of phenotype descriptions** so that character analysis and character construction no longer represent a black box or remain to be a matter of authority. We can develop algorithms that **quantify** the **degree of similarity** between two given descriptions, hence subjecting similarity propositions to constructive criticism and corrections and providing comparative phenotypic methods a **mathematical statistical analytical framework** [29]. Eventually, the semantic framework could provide the unified theory of character construction that biology is yet lacking [61].

In the following, we discuss two different approaches to semantically representing phenotype descriptions. We argue that, from a conceptual point of view, the Phenotype Knowledge Graph approach is superior to the Semantic Phenotypes approach because it minimizes the number of TBox expressions necessary for carrying descriptive contents, which brings about the technical advantage of each entity, quality, and relation being referred to in a description having its own URI. As a consequence, these entities, qualities, and properties can be individually identified, which in turn brings about various practical advantages that together better meet the above-mentioned requirements.

## Results

### Semantic phenotypes: phenotype descriptions as class-based TBox representations

When researchers started to conduct large-scale mutagenesis screens in model organisms, labs were suddenly able to analyze large collections of mutants. This raised new challenges regarding scale and complexity of the newly generated data and their analysis and interpretation with respect to their relationship to corresponding phenotypes, leading to the use of ontologies for standardizing mutant phenotype descriptions [56, 62–64]. These mutant phenotype descriptions were **comparative phenotype descriptions**, i.e., phenotype descriptions that are based on comparative observations that characterize the outcomes of an experiment or observed difference against a specific reference state such as the mutant in comparison to the wild-type [65]. The problem with comparative descriptions against some ‘normal’ condition or state is that they describe *instance anatomy* in reference to *canonical anatomy* and in doing so convey information about at least two different entities, i.e., a particular mutant and a ‘universal’ wild-type. This restricts the usability of the descriptions to the context of comparison against the wild-type, because the direct observation, on which the comparison is based, often cannot be derived anymore. When for instance stating that the described specimen has an *“increased length of abdomen”*, we do not know the actual length of the specimen’s abdomen, not even relatively as in *“length of abdomen above 2.6 mm”*. If we want to get any information about that abdomen, we must first consult the description of the wild-type, in order to derive the lower boundary value of the possible length for the described abdomen.

With respect to the application of ontologies for standardized mutant-phenotype descriptions, two different class-based approaches were initially followed [64, 66–68]:

1. The **class-based pre-composition approach** uses a single dedicated ontology that provides phenotype descriptions in the form of ontology classes for annotating natural language descriptions of phenotypes, with each phenotype having its corresponding ontology class [53] (*pre-composed*, because the phenotype description is completely covered by the definition of the respective ontology class; reference to the URI of that ontology class is sufficient). The definitions of phenotype classes usually reference a combination of entities and values (e.g., abnormal body weight, id MP:0001259, of the Mammalian Phenotype ontology, MP). A particular phenotype is then described by referencing the URI of a corresponding ontology class [65]. In order to be able to reference a suitable URI, however, the ontology class must be pre-composed in advance by the ontology editor of the respective phenotype ontology (see *pre-composition* [69]).
2. In 2004, the need for a more systematic and formalized approach was recognized, resulting in the **class-based post-composition approach** and the development of the Phenotype And Trait Ontology (PATO) [70], a species-neutral ontology of attributes and values [65]. The post-composition approach characterizes and defines phenotypes following a formalized syntax using class expressions from various distinct ontologies and applying the entity-quality (EQ) format [71, 72] (*post-composed*, because the actual phenotype description must be composed from references to several ontology classes and/or values using the EQ format). According to this post-composition approach, one characterizes a phenotype in terms of a bearer entity (E) that is described by a specific quality (Q). The term that defines the bearer entity is provided by a class of some domain ontology, the term defining the specific quality by a class of PATO. The resulting EQ statement completely replaces the natural language description of the phenotype. Originally, the statement took the tripartite structure of *Entity* + *Attribute* + *Value* such as in eye + color + red (*EAV* or *Entity-Attribute approach* [66, 71, 72]). After the quality terms in PATO have been organized hierarchically, with more specific terms such as red (id PATO:0000322) being subsumed as subclasses under more general terms such as color (id PATO:0000020), the tripartite structure has been adapted to the bipartite structure of *Entity* + *Quality* [48, 50, 51, 56, 63, 73–76]. As a result, the natural language phenotype description *“eye has red color”* translates into the EQ statement eye + red, with, for instance, eye (id MA:0000261) from the Mouse Adult Gross Anatomy Ontology and red (id PATO:0000322) from PATO.

An obvious advantage of the post-composition approach is that it limits the number of ontology terms required for describing a given phenotype because annotators have the ability to compose phenotype descriptions on-the-fly using combinations of terms from available ontology classes to form a multiplicity of different EQ statements [67, 69]. The bipartite structure of the EQ statements also lends itself for being stored in a table of a relational database, with E and Q each providing a value in the form of a URI for a corresponding cell in the table. However, phenotype annotations in such tables must not be confused with representing phenotypes as semantic graphs, because these URIs only have the function of providing semantic links for the E and the Q of an EQ statement from a table to the corresponding ontology classes of ontologies. When an EQ statement is stored as a set of URIs in a table in a relational database, the link between the URI in the E position and its corresponding URI in the Q position is provided implicitly through the position of their cells within the database table, but it is not explicitly stated like it is when representing the EQ statement as a TBox semantic graph.

**Fig. 2:**
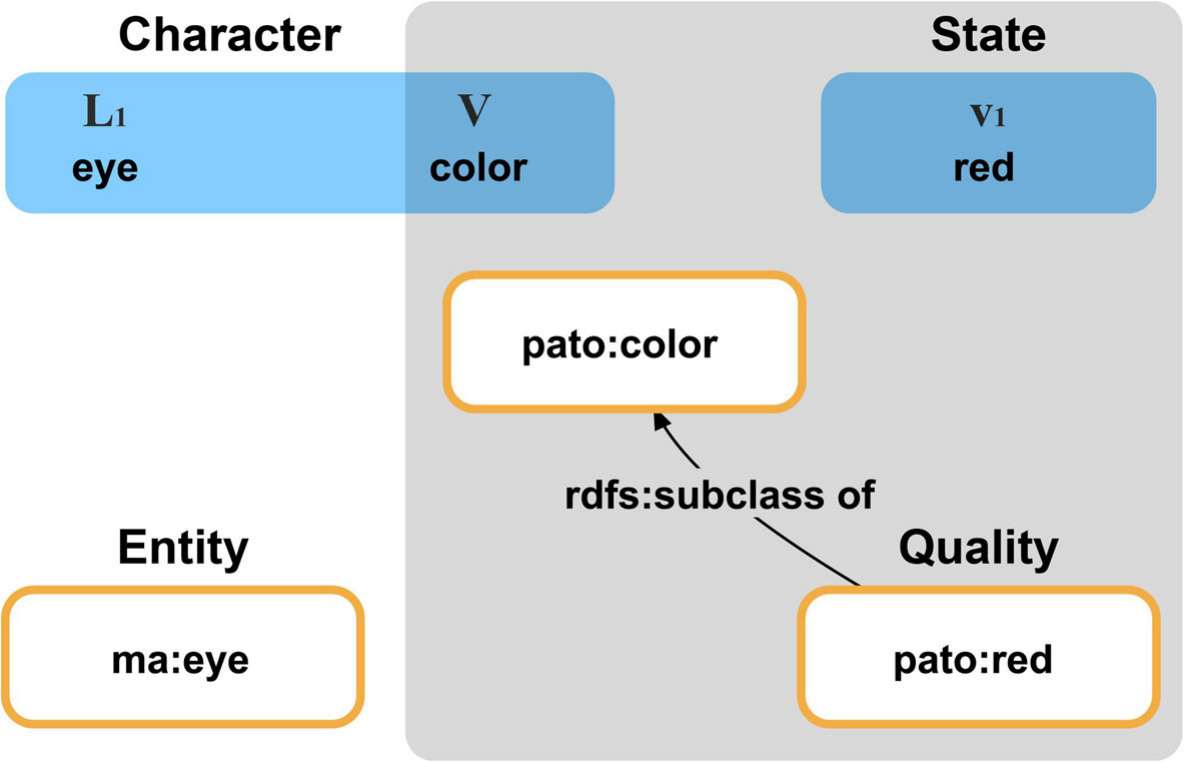
A phylogenetic character statement compared to a phenotype description based on the EQ model. The upper line represents the character statement *“eye color: red”*, following the syntax suggested by Sereno [77], with **L**_**1**_ indicating the (first) locator and **V** the variable, which together constitute the character part of a character statement. The character state is represented by the value **v**_**1**_, which is one of the possible states defined for the character statement. This phylogenetic character statement can be translated into the EQ statement eye+red, with eye from, e.g., the Mouse Adult Gross Anatomy Ontology (id MA:0000261), representing the locator **L**_**1**_ part of the character and red from PATO (id PATO:0000322) representing the value **v**_**1**_ of the character state. Because PATO organizes quality terms in a nested hierarchy of increasingly differentiated attributes, the reference to red implicitly references also color (id PATO:0000020) and thus the variable **V** part of the character (cf. [52])

Initially, the EQ format was used for characterizing and classifying different mutant phenotypes of a given model organism by comparing them to their canonical wild-type and then relating them to their underlying genotype [56, 64, 73, 74]. The wild-type functioned as a ‘normal’ condition and point of reference. Respective phenotype descriptions thus contained comparative phenotype statements [65] that describe *instance anatomy* in reference to *canonical anatomy*. However, the EQ approach was soon picked up by evolutionary morphologists, who modified it to describe characters and character states as they are known from phylogenetic character matrices, resulting in **direct phenotype descriptions** [65] that allow describing phenotypes in the framework of *instance anatomy*. Typical character and character state descriptions such as *“eye color: red”* would lend themselves to being translated into EQ statements, with Q representing the character state (Fig. 2) [52, 56, 63, 69].

Many phylogenetic characters, however, cannot be translated into the strict EQ syntax and require modifications [50]. Composite characters, for instance, require the reference to more than one entity or quality term, extending the EQ statement to a nested composition, in which the E and/or the Q of the phenotype description are themselves represented as one or several EQ statements. Relational characters require modifying the EQ model to E (QRE) (RE = related entity) and quantitative characters to E (QC) (C = count) (see, e.g., Phenoscape’s Guide to Character Annotation).

When an EQ statement is stored in a relational database, with the URIs of ontology classes providing only semantic links between the E or the Q of the statement and the definitions of the corresponding ontology classes, additional coding is required to convert the table into an OWL file that documents the EQ statement as a semantic graph. Similarly, EQ statements stored within NeXML files [78, 79], an XML-based phylogenetic data exchange standard inspired by NEXUS (e.g., [50]), as used by the Phenoscape project, require separate software for conversion to a semantic model in OWL.

Although at first glance the class-based pre-composition and the class-based post-composition approaches appear to be incompatible, it has been demonstrated that they are actually complementary and fully compatible because single term expressions and (composite) EQ statements can be related to each other as being equivalent, providing each pre-composed ontology class with a possible equivalent logical definition in the form of a corresponding EQ statement [69]. Any given EQ description of a phenotype can thus be translated to the definition of a corresponding ontology class (i.e., Semantic Phenotype expression class) that represents that specific phenotype, and vice versa [48, 67]. As a consequence, EQ statements can be described within ontologies, documented and exported as OWL files, and represented as class-based TBox semantic graphs.

Taxonomists became interested in the OWL-based documentation of phenotypes and suggested that the EQ approach could also be used for taxonomically describing phenotypes [51, 75]. Respective EQ statements can be composed as axioms of corresponding ontology classes with the help of ontology editors such asProtégé [80], using OWL Manchester Syntax [60] and following general composition schemes [48, 51, 81, 82]. In order to express that a particular specimen bears a specific phenotype, the specimen is represented in OWL as an individual resource with its own URI. This individual is specified to be an instance of the ontology class that defines the phenotype. The resulting direct phenotype descriptions have been called **Semantic Phenotypes** and each Semantic Phenotype is attached to one (or more) particular specimen [51, 75, 81–83]. Semantic Phenotypes, thus, can be completely expressed in OWL and stored in a separate OWL file [51].

In some sense, Semantic Phenotypes combine the class-based pre-composition approach with the class-based post-composition approach since the description of Semantic Phenotypes first requires the definition of the corresponding phenotype classes (pre-composition), which in turn use ontology classes in their axioms (post-composition).

Direct anatomical phenotype descriptions are essentially ‘factual’ anatomical descriptions consisting of assertional statements that document empirical observations about particular anatomical entities. A Semantic Phenotype represents the ‘factual’ anatomical description through a single ABox expression that specifies a phenotype class that is instantiated by the ODU. All the actual descriptive content is implicitly contained in the referenced phenotype class in the form of class axioms and thus TBox expressions. Consequently, the amount of required TBox expressions in the description exceeds the necessary minimum. This represents a conceptual choice that can be traced back to the history of the Semantic Phenotype approach, which applied semantic workflows and tools that originated from mutagenesis research on model organisms (i.e., *Homo sapiens* and others) and were therefore conceptualized for comparative phenotype descriptions that reference to *canonical anatomy*. While this choice has **technical implications**, as long as phenotypes are only annotated within relational database tables using sets of URIs of ontology classes instead of documenting them as semantic graphs in a knowledge base, the technical implications have no practical consequences.

The most important technical consequence of documenting descriptive contents using TBox expressions is the fact that all entities, related entities, and qualities mentioned in the axioms of phenotype ontology classes used in Semantic Phenotypes are anonymous resources and therefore do not possess their own URIs. They cannot be referenced individually, and more complex Semantic Phenotypes cannot be easily partitioned into simpler descriptive fragments. This has far-reaching consequences that also affect reasoning over Semantic Phenotypes, requiring the implementation of additional rules to relate anonymous resources with one another and with the specimen they describe. For instance the phenotype “antenna longer than eye” can be expressed in OWL Manchester syntax as ‘has part some (antenna and *bearer of* some (length and *increased in magnitude relative to* some (length and *inheres in* some eye)))’ (‘and’ being used in the sense of intersection of two sets and ‘some’ in the sense of the existential quantifier ‘there exists a’ or ‘some instance of’), with the consequence that ‘some antenna’ and ‘some eye’ are anonymous resources so that *“to be an instance of this class, an antenna needs to merely be longer than at least one eye in the world, not necessarily an eye possessed by the same organism”* ( [51], p. 643).

### Phenotype knowledge graphs: phenotype descriptions as instance-based ABox representations

Another approach for using ontologies to standardize **direct phenotype descriptions** has been suggested that represents particular phenotypes as instance-based ABox semantic graphs, called **Phenotype Knowledge Graphs** [47, 49, 57]. Phenotype Knowledge Graphs can be stored in separate OWL files and take the form of ‘factual’ anatomical descriptions. Contrary to Semantic Phenotypes, Phenotype Knowledge Graphs refer to particulars for the description of a given phenotype and thus minimize the amount of required TBox expressions to the class specifications that each identified part of the ODU instantiates, while describing the particular qualities of the parts and the actual relationships between them as ABox expressions, instead of describing them through class axioms. In other words, each anatomical entity, quality, and property described in a Phenotype Knowledge Graph is represented as a particular that possesses its own URI and that instantiates a corresponding ontology class, which in turn is necessarily specified using TBox expressions. As a consequence, each described part, quality and property can be individually referenced and identified through its own URI.

**Fig. 3:**
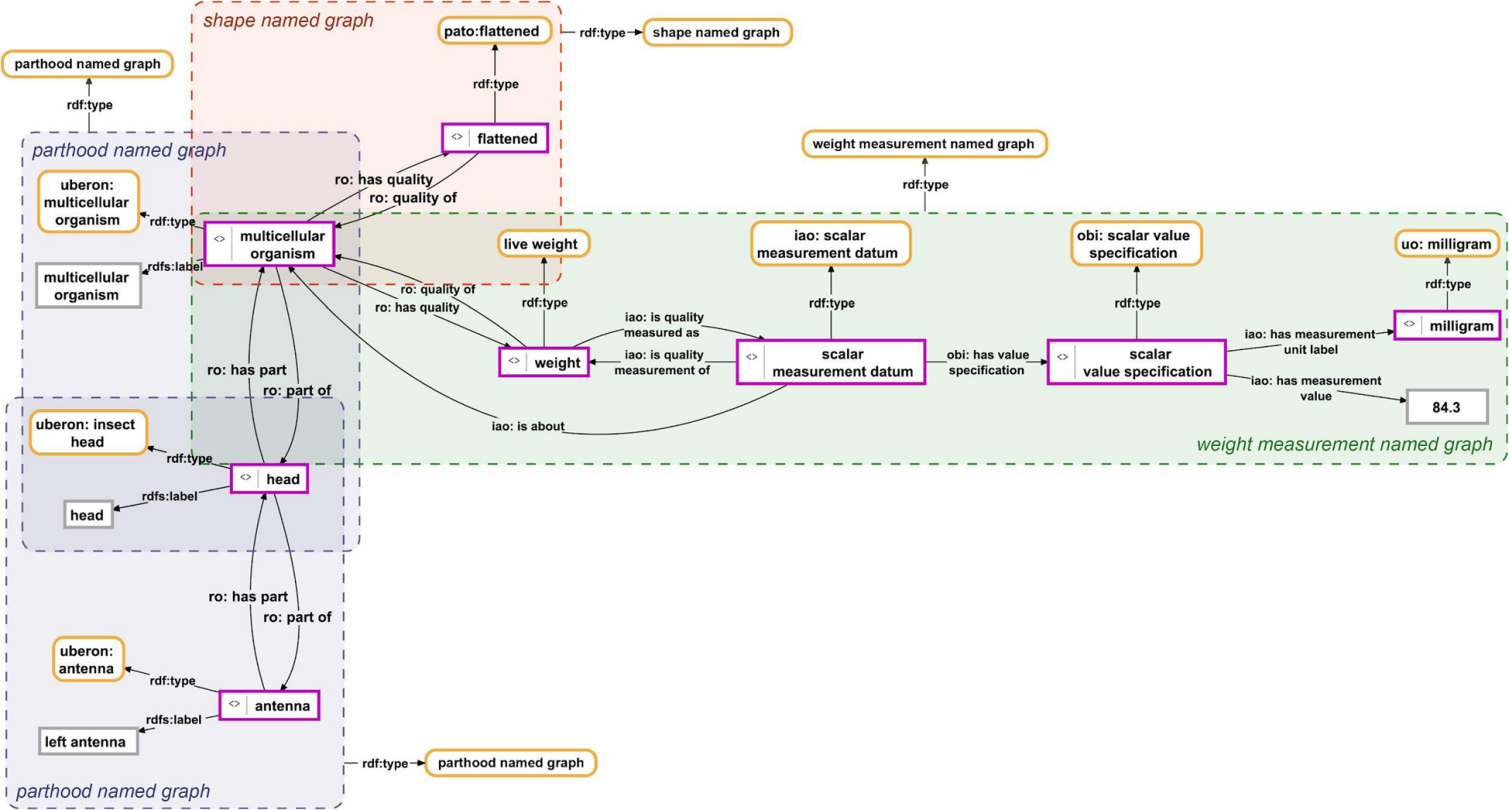
Phenotype Knowledge Graph. The instance-based ABox semantic graph shows the description of a multicellular organism. It consists of instances (purple-bordered boxes), each of which instantiates a specific ontology class (yellow-bordered boxes with rounded corners) through the *type* property. All instances referring to anatomical entities possess a human-readable label (grey-bordered box connected through the *label* property) and are connected via parthood relations, forming a partonomy. The partonomy indicates that this instance of multicellular organism possesses an instance of insect head that, in turn, possesses an instance of antenna. The multicellular organism instance is further described to have a flattened shape and a measured live weight of 84.3 milligrams. The semantic graph is organized and fragmented into different subgraphs, each of which is contained in its own named graph (dashed-bordered colored boxes). Each subgraph contains information relating to a specific perceptual question that can only be answered empirically. In other words, each named graph contains a separate empirical observation. And each named graph also possesses its own URI and instantiates a named graph ontology class. The Phenotype Knowledge Graph is the union of all the named graphs

When describing an ODU following the Phenotype Knowledge Graph approach, one first must decompose the ODU into the constituent parts one wants to cover in the description. Each part belongs to a specific kind of anatomical entity and is therefore represented as an instance of the corresponding ontology class. All the described parts are related to one another through parthood relations. The resulting parthood hierarchy provides the organizational backbone for a Phenotype Knowledge Graph and is in that function comparable to the taxonomy (i.e., class-subclass hierarchy) of classes of an ontology [57]. Next, one can describe each constituent part in more detail, specifying its various properties and qualities, including the specification of relations between parts (Fig. 3).

By referencing the ontology classes that are instantiated by described parts, Phenotype Knowledge Graphs link to one or more ontologies, which allows applications that process and analyze Phenotype Knowledge Graphs to utilize not only the information contained in the descriptions themselves (*instance anatomy* data) but also the information contained in all referenced ontologies (*canonical anatomy* and thus *invariant knowledge*). The combined information can be used for inferencing and quantitatively comparing different Phenotype Knowledge Graphs [29, 58, 59].

Phenotype Knowledge Graphs can be meaningfully fragmented into several flexibly manageable subgraphs, with each subgraph corresponding to a specific type of descriptive statement [57]. For example, the parthood relation between two anatomical entities, the shape specification of a particular anatomical structure, or the specification of its weight measured in milligrams can be associated with its own named graph resource (Fig. 3). A named graph resource is a URI that identifies a set of triple statements by adding this URI to each triple belonging to the named graph, thus turning the triples into quads. Phenotype Knowledge Graphs can be organized into named graphs, stored in a tuple store, and be made accessible through a SPARQL^12^ endpoint [84].

Phenotype Knowledge Graphs express phenotype descriptions wherever possible as assertional statements in the form of instance-based ABox semantic graphs, requiring only a minimum amount of TBox expressions. Consequently, the Phenotype Knowledge Graph approach does not suffer from the technical implications resulting from expressing phenotype descriptions as universal statements using the EQ format. In the following, we discuss these technical implications and evaluate their practical consequences based on the technical requirements for empirical data mentioned above and their relation to eScience-compliant data and metadata standards and the FAIR Guiding Principles.

## Discussion

Before we discuss the technical advantage of the instance-based ABox approach and its practical implications, we want to emphasize once more that the here discussed limitations of the TBox approach apply in the context of **documenting empirical data and metadata in a knowledge graph**. There are many other contexts, in which TBoxes can be superior to ABoxes. For instance, when documenting or using **invariant knowledge** (see 104) and thus universal statements instead of assertional statements, where ABoxes cannot be used. In anatomy, this would relate to the context of *canonical anatomy*.

When reasoning over your data is important, TBoxes may in some cases also be superior to ABoxes. However, whereas, reasoning has primarily been applied for validating the consistency of class hierarchies and for inferring additional subsumption relationships [85], the need for reasoning over ABoxes has been identified by now and corresponding reasoners such as Arachne [86] are being developed that support reasoning on, e.g., property relationships. Reasoners such as ELK, that are commonly used with TBoxes, use the OWL EL profile, which does not support ABox reasoning very well. Arachne uses the OWL RL profile, which is better suited for instance data. Arachne can, e.g., be used when adding an ABox to a knowledge graph for suggesting additional inferred statements and for checking for consistency in real time―TBox reasoners such as ELK are well suited for tasks like ontology classification and consistency checking of ontology classes, but do not perform well for real-time multi-user online systems focused on ABox graphs, because they do not support axioms like inverse properties, property ranges, and materialization of object property assertions [86]. When having to compare an actual state of a system, as it can be recorded, e.g., via sensors and documented as an ABox, against a target state, which could be an established standard documented as a TBox, you can check the ABox for consistency against the TBox using Arachne.^13^

Due to the tabular architecture of relational databases, TBoxes have an advantage over ABoxes when storing data in a relational database, because assertional statements can be documented as instances of ontology classes that, in turn, follow the EQ or EAV model and provide the description of the actual content in their class axioms. Therefore, one only has to store the URI of the ontology class as a value in a respective table to document the content specified through that class’s axioms.

The choice of whether to use a relational database or a knowledge graph for storing, documenting, and managing research data should be driven by the requirements of your study or project and the competency questions that you derive from your respective user stories. Relational databases are well suited for closed world systems, for which you can specify the data schema before populating your database with data, whereas knowledge graphs are well suited for open world systems and thus systems that assume incomplete knowledge by default, where you can easily extend the data schema on-the-fly. Also, (i) if the query structure is well known and expected to be stable―you know, which questions the dataset has to answer and these questions will not likely change in the future, (ii) if you know that the dataset may grow, but only the same type of data will be added, or (iii) if your dataset is not complex and its data points are not heavily interconnected so that it can be easily represented in the tabular structure of a relational database, relational databases may be superior to knowledge graphs as a technical solution for your data management.

In the following, we discuss the technical difference between semantic phenotypes and phenotype knowledge graphs as examples for the class-based TBox and the instance-based ABox approach and the practical implications of this difference in the context of documenting empirical data and metadata in a knowledge graph. 

### Decomposing phenotype descriptions into separate observation-based statements

Unlike Semantic Phenotypes, Phenotype Knowledge Graphs can be fragmented in various ways into meaningful subgraphs. As a consequence, they provide significantly more flexibility in what can be done with them. Each subgraph can be organized in its own particular named graph that possesses its own URI (see Fig. 3). Each named graph resource can be associated with a corresponding ontology class that it instantiates. These classes can be defined in a domain reference ontology for anatomy that specifies a semantic data model for anatomy [57]. In this way, one could define an ontology class for each type of descriptive statement relevant for phenotype descriptions. Each class defined this way can be understood to correlate with a specific perceptual question that can only be answered by studying the relevant parts of the given ODU. The respective question thereby functions like a **perceptual category** that is part of a general phenotype structure concept [8, 47, 49]. Examples for such questions would be: What is the weight of this anatomical structure? What is the length of this anatomical line? What is the volume of this anatomical space? What is the position of this anatomical point? What is the color of this anatomical surface? What is the general shape of this anatomical structure? What is the biological function of this anatomical structure? From which structure did this anatomical structure develop?

Each named graph belonging to a phenotype description refers to the combination of (i) a particular part of the ODU and (ii) a specific perceptual category. Fragmenting a given phenotype description into several such named graphs can be understood as the decomposition of the description into its **smallest units of empirical information** and thus into a set of particular descriptive statements. As a consequence, any given Phenotype Knowledge Graph can be fragmented into its descriptive statements in the form of subgraphs and these subgraphs can be united again to return the Phenotype Knowledge Graph. This general approach is not restricted to anatomy and can be applied to any empirical data.

The **decomposability of Phenotype Knowledge Graphs** in particular and of instance-based ABox semantic graphs in general **is the most important technical difference** compared to Semantic Phenotypes and class-based TBox semantic graphs and has significant consequences that substantially affect various practical aspects.

#### The explorability of phenotype descriptions

Based on the ontology classes of descriptive named graphs discussed above, one can flexibly define various **data views** for **exploring** Phenotype Knowledge Graphs [57]. Each data view is defined in reference to one or more such classes. One data view could, for instance, be defined in reference to the class of weight measurements, whereas another one could comprise all classes that contain measurements in general. Applying the former data view on a given description would result in the union of all subgraphs of the description that contain weight measurement data, whereas the application of the latter would result in the union of all subgraphs containing measurement data in general. The definition of various such data views would significantly improve the possibility to **meaningfully navigate** semantic graphs of phenotype descriptions **without** users of respective applications having to write deeply nested **SPARQL** queries, because only the corresponding named graphs must be identified. This, again, applies in general to all kinds of empirical data that are represented as instance-based ABox semantic graphs.

Unfortunately, Semantic Phenotypes and any other class-based TBox semantic graph cannot be fragmented this way, because the entities, related entities, and qualities that class axioms refer to are anonymous resources and thus cannot be individually referenced and identified (see above). Therefore, Semantic Phenotypes cannot be explored to the same degree as Phenotype Knowledge Graphs.

#### Linking relevant metadata and supplementary contents to phenotype descriptions

**Fig. 4:**
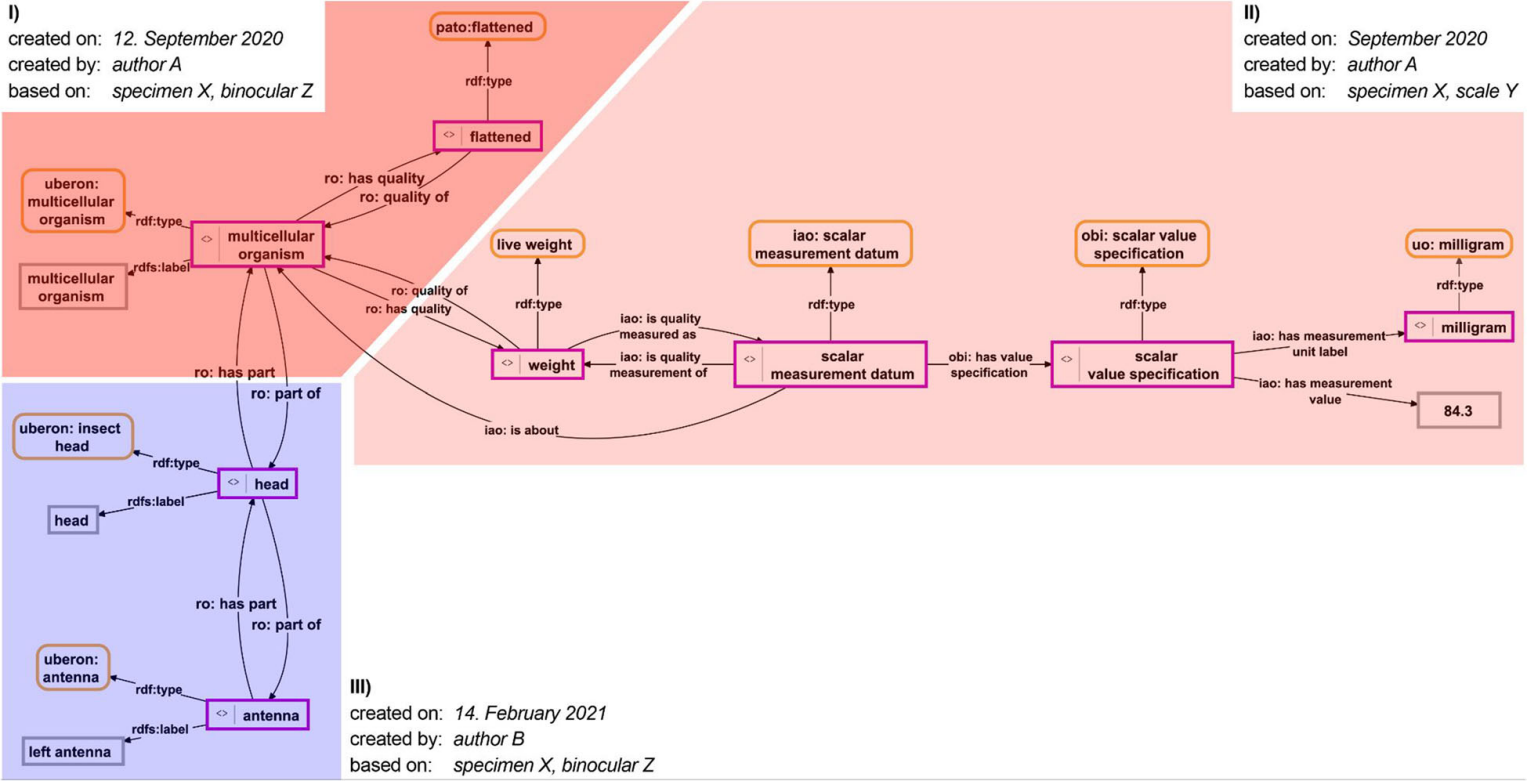
Metadata about a Phenotype Knowledge Graph. The Phenotype Knowledge Graph from Fig. 3 with its associated metadata. The Phenotype Knowledge Graph is organized into three subgraphs (I, II, and III), each of which has its own set of metadata statements associated. When compared to Fig. 3, one can identify the three subgraphs in reference to named graphs: subgraph I) refers to the ‘shape named graph’, subgraph II) to the ‘weight measurement named graph’, and subgraph III) to the union of the two ‘parthood named graphs’

Metadata are statements about statements. In the case of phenotype descriptions, metadata refer to who contributed which parts of the description, based on which evidence, and using which instruments, where and when (see Fig. 4). Modeling statements about statements within OWL/RDF is not trivial and various approaches have been suggested [87]. OWL itself provides the possibility to make statements about statements using standard reification, by specifying the statement about which one wants to make statements through three additional triple statements (i.e., statement_URI *subject* subject_URI; statement_URI *predicate* predicate_URI; statement_URI *object* object_URI). While this may be a practical solution for making statements about a single triple statement, it becomes very impractical if one has to make statements about a subgraph that consists of several triple statements (see example Fig. 4). For such cases, the use of named graphs is a good choice. Moreover, named graphs also outperform other metadata representation models when conducting more complex queries [87].

Because each descriptive statement belonging to a Phenotype Knowledge Graph is organized in its own particular named graph and this named graph has its own URI, it can be individually referenced for associating relevant **metadata information** to it, such as on which specimen the observation is based, which microscope has been used or the literature source from which the information in that subgraph of the description has been taken and how reliable that source is [57]. Each such metadata, in its turn, can be documented in its own named graph and thus be clearly separated from the actual description. The combination of a particular descriptive named graph and its associated metadata named graph can be published separately from the whole description as a **nano-publication** [88–90].

Moreover, by referring to the URI of the particular named graph, one can also link natural language descriptions and semantically annotated media contents to each descriptive statement, as well as comments and other annotations. And because each described part, quality, and property possesses its own URI in a Phenotype Knowledge Graph, images can be annotated with regions of interest using these URIs to indicate that they depict a particular part, quality or property, which is not possible with Semantic Phenotypes.

As a consequence, the use of the description named graphs allows for differentially assigning metadata, unstructured natural language texts, and media contents at the level of smallest units of semantically meaningful empirical information contained in a Phenotype Knowledge Graph instead of having to assign them to the description as a whole, and this information can be published as a **micro-publication** [91]. And again, this is not restricted to the domain of anatomy, but can be applied to all kinds of empirical data that are represented as instance-based ABox semantic graphs.

Unfortunately, Semantic Phenotypes and any other class-based TBox semantic graph cannot be fragmented this way and thus assigning metadata, natural language texts, and media contents at the level of smallest units of empirical information is not that straight forward.

#### Expandability of phenotype descriptions

It is impossible to describe a given specimen covering all aspects that could be relevant. Like any other description of a particular material entity or process, each phenotype description represents a decomposition that is based on a **virtual partition** of the ODU into the parts that are relevant for the specific frame of reference applied by the person making the description [92–94]. Due to the phenotypic complexity of anatomical entities, which often covers several levels of granularity, ranging from the molecular level to the cellular level and the level of gross anatomy, descriptions of specimens are never complete, irrespective of the applied frame of reference. This applies to Semantic Phenotypes in the same way as to Phenotype Knowledge Graphs. The problem of the **incompleteness of phenotype descriptions**, however, confronts the Semantic Phenotype approach and the class-based TBox semantic graphs in general with a conceptual dilemma. If a given Semantic Phenotype must be complemented with additional information, resulting in a more detailed representation of the described phenotype, one can choose between:

1. Defining a new phenotype class that incorporates all information of the original phenotype class and, additionally, also covers the new information. The new phenotype class then replaces the original class and the new Semantic Phenotype the original Semantic Phenotype. This, however, would not only result in increasingly complex axiom expressions, which become increasingly incomprehensible, but tracking provenance and all relevant metadata across the different versions will be problematic as well, especially since Semantic Phenotypes cannot be easily fragmented.
2. Defining a new phenotype class that only covers the additional information. The corresponding Semantic Phenotype would complement the original Semantic Phenotype. This is also problematic since the parts and properties mentioned in the class axiom of the original phenotype class cannot be referenced in the complementing phenotype class, because they are anonymous resources. As a consequence, the complementing Semantic Phenotype will, for instance, describe in more detail one of the parts mentioned in the class axiom of the original phenotype class, but the original and the complementing Semantic Phenotype graphs will not connect due to the anonymity of the described parts.

Phenotype Knowledge Graphs and instance-based ABox semantic graphs in general, on the other hand, can easily be expanded with additional information. Because each described part, property, and quality possesses its own URI, existing descriptions can be easily expanded through nano-publications and their corresponding metadata be tracked independently of the metadata of the original description.

#### Integrating different frames of reference in a phenotype description

As mentioned above, any given phenotype can be described from different **frames of reference**, e.g., from a purely spatio-structural, a functional, or a developmental perspective. Each frame of reference will likely virtually partition the underlying ODU in its own particular way. Descriptions of the same phenotype that are based on different frames of reference thus often result in incongruent partitions [94]. As a consequence, the representation of a phenotype through a single phenotype ontology class will make it very difficult to cover all information relevant to the various frames of reference relevant in the life sciences because the corresponding class axiom can only model one of the many possible virtual partitions. In other words, a purely spatio-structural description of a given phenotype must be represented with a different phenotype class then a functional, a developmental, or an evolutionary description of that same phenotype. This would result in a spatio-structural Semantic Phenotype, a functional Semantic Phenotype, a developmental Semantic Phenotype, and an evolutionary Semantic Phenotype, each of which would refer to the same given ODU. Due to the problem of anonymous resources, even if each of these descriptions would refer to the same part in the ODU, the resulting graphs would not connect because this part would be represented as anonymous resources.

With the Phenotype Knowledge Graph approach, on the other hand, any given phenotype can be described in reference to a specific frame of reference and the resulting graph will connect spatio-structural descriptions of a given described part with its functional, developmental, and evolutionary descriptions, because this part possesses its own URI and thus can be referenced in any possible virtual partition of a given ODU. Contrary to the Phenotype Knowledge Graph approach, the Semantic Phenotype approach with its class axioms seems to be not well suited for integrating different frames of reference in a given phenotype description.

#### The open world assumption and the need for negations and specifications of quantities of parts

No ODU can be comprehensively described across all possible frames of reference, scales, and granularity levels. No semantic representation of an ODU can be exhaustive in that respect. Any ODU possesses a virtually infinite number of possible partitions so that no phenotype description can be considered to cover all of them. This situation is dealt with by the so-called **Open World Assumption (OWA)**. OWA assumes incomplete information by default. A direct consequence of OWA is that the lack of knowledge about a fact does not immediately imply knowledge of the negation of that fact. This means, for instance, that when a description does not state that a particular insect head has cells as its parts, we cannot conclude that the head is not composed of cells.

OWL and description logics-based ontologies adhere to OWA by default, and so do both the Phenotype Knowledge Graph and the Semantic Phenotype approach. In both approaches, when starting to describe an ODU, everything is considered to be possible. This space of possibilities becomes more and more constrained and restricted with the addition of information. Following this notion, phenotype descriptions restrict what is possible [58].

**Fig. 5:**
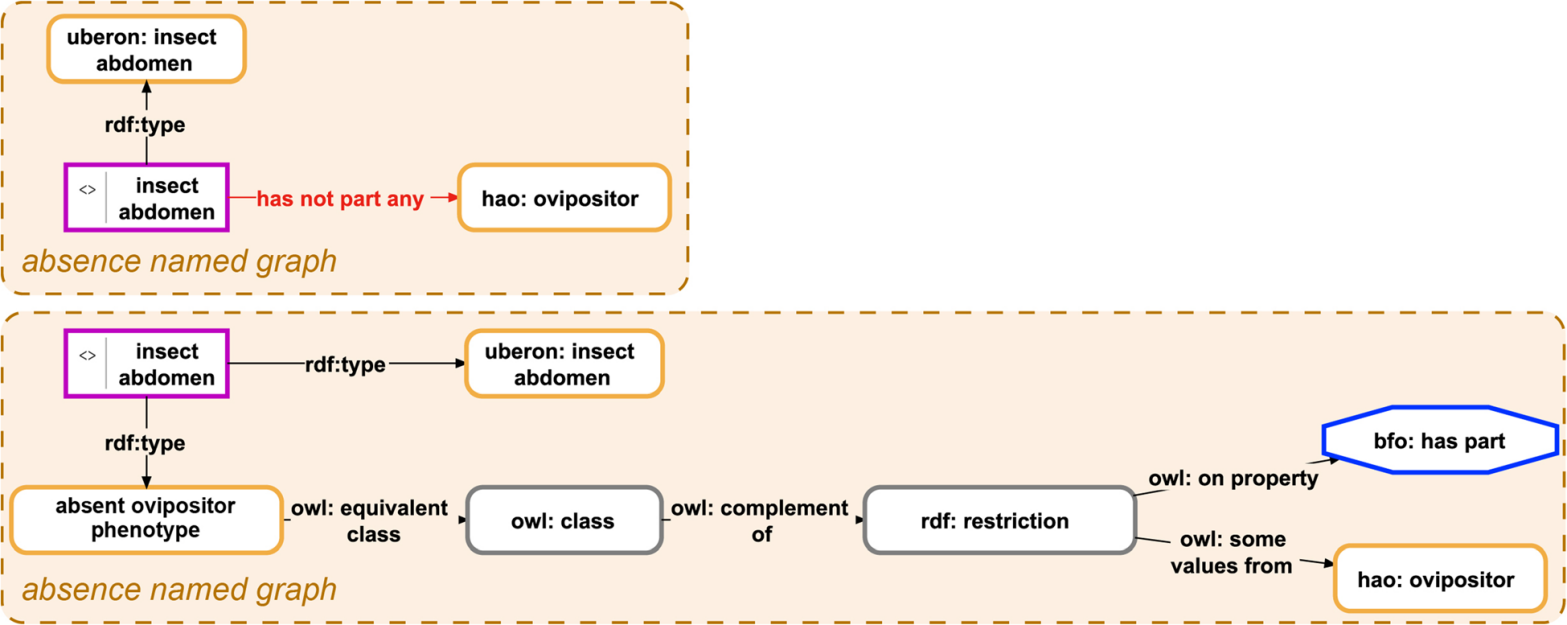
Two alternative models for documenting absences using the instance-based ABox semantic graph approach. Within the Phenotype Knowledge Graph approach, the observation *“insect abdomen lacks an ovipositor”* can be modeled in two alternative ways. **Top**: Shows a representation of the observation using only ABox expressions. This requires the introduction of the object property *has not part* any that has an instance as a domain restriction and a class as range restriction. This is not part of the OWL syntax and would require the introduction of additional tools for making it machine-actionable. **Bottom**: Shows a representation of the observation using a combination of ABox and TBox expressions. The instance of insect abdomen instantiates not only the class insect abdomen but also the class absent ovipositor phenotype, which is characterized as the complement class of the class of entities that have some ovipositor as their part. This description is compliant with description logics and is directly machine-actionable. *Purple-bordered box = instance resource; yellow-bordered box with rounded corners = ontology class resource; grey-bordered box with rounded corners = anonymous class; blue-bordered octagon = object property class; labeled arrow = property resource*

**Fig. 6:**
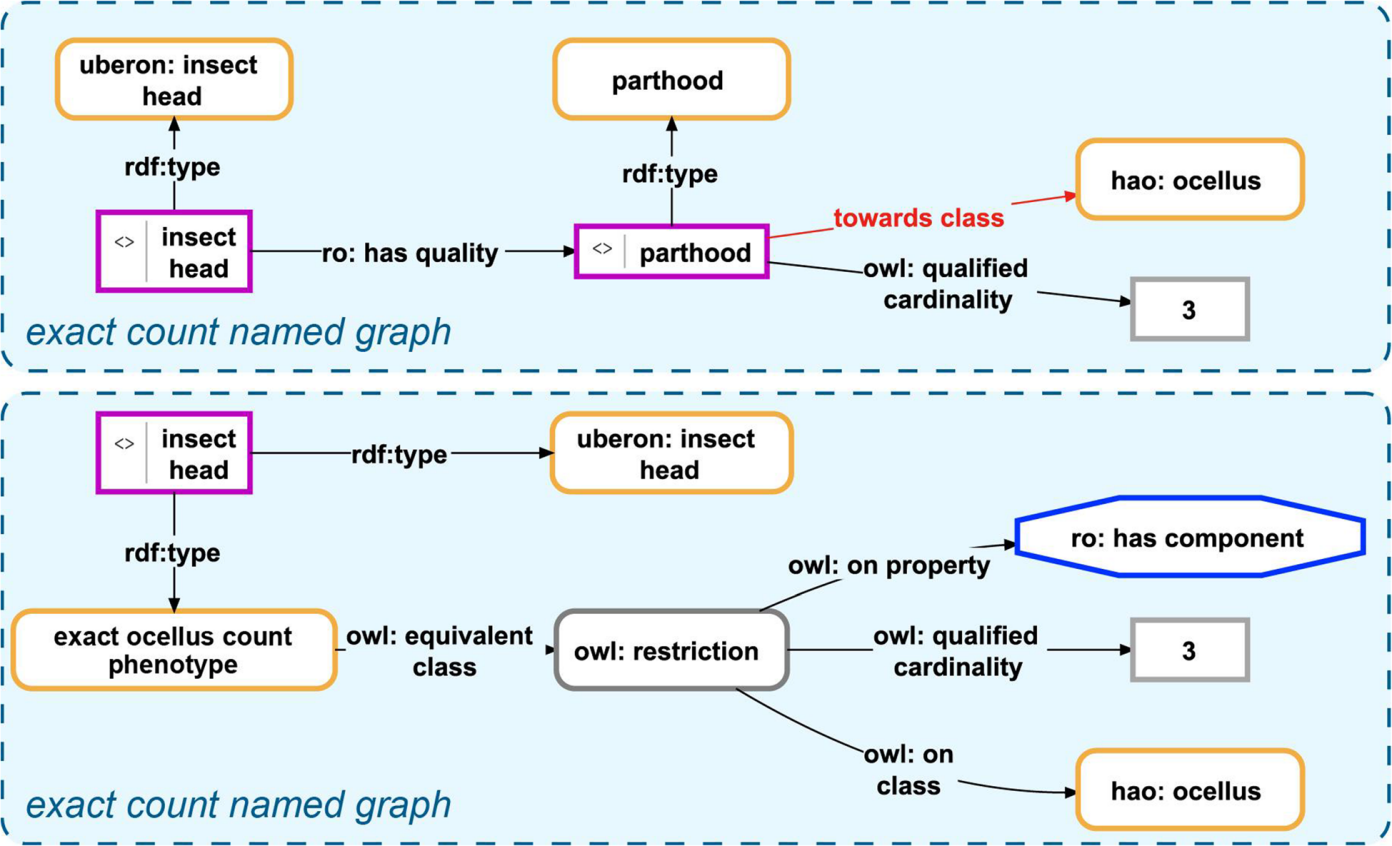
Two alternative models for documenting exact counts of parts using the instance-based ABox semantic graph approach. Within the Phenotype Knowledge Graph approach, the observation *“insect head has part exactly 3 ocelli”* can be modeled in two alternative ways. **Top**: Shows a representation of the observation using only ABox expressions. This requires modeling the parthood relation as a directed relational quality and as a consequence of that the introduction of an object property *towards class* that has an instance as a domain restriction and a class as range restriction. Unfortunately, modeling the observation this way is not compliant with description logics and would require the introduction of additional tools for making it machine-actionable. **Bottom**: Shows a representation of the observation using a combination of ABox and TBox expressions. The instance of insect head instantiates not only the class insect head but also the class exact ocellus count phenotype, which is characterized as a cardinality restriction on a combination of a property and a class. This description is compliant with description logics and is directly machine-actionable. *Purple-bordered box = instance resource; yellow-bordered box with rounded corners = ontology class resource; grey-bordered box with rounded corners = anonymous class; grey-bordered box with sharp corners = value; blue-bordered octagon = object property class; labeled arrow = property resource*

OWA is not problematic for phenotype descriptions per se. It for instance allows reusing and extending phenotype descriptions, adding more information to already existing descriptions whenever necessary. But in some cases, we want to make clear that a given ODU possesses, e.g., only two antennae and lacks an ovipositor―information that cannot be provided by describing only two antennae and not describing any ovipositor. While one could introduce specific properties to model such information as instance-expressions (see Fig. 5, top, and Fig. 6, top), any such model will not be compliant with description logics and could therefore not be reasoned on. Making these expressions machine-actionable would thus require additional efforts. Alternatively, one can describe this type of information with the help of class-expressions and thus TBox expressions, using OWL Manchester Syntax. The observation *“insect abdomen lacks an ovipositor”* translates to the Manchester expression ‘not ( *has part* some ovipositor )’ and the observation *“insect head has part exactly 3 ocelli”* to ‘*has component* exactly 3 ocellus’.^14^ Both Manchester expressions can be represented as class-based semantic graphs and be used within the Semantic Phenotype approach as well as the Phenotype Knowledge Graph approach (see Fig. 5, bottom, and Fig. 6, bottom).

#### Demarcating units of description

Another problem with Semantic Phenotypes is whether a given specimen should be described using a single complex Semantic Phenotype or a set of multiple Semantic Phenotypes. Should a phenotype be defined in a single phenotype ontology class or in several such classes? Should the unit of description equal the smallest unit of semantically meaningful empirical information? In the end, it is the question of what is the criterion for demarcating units of description [47]? And again, part of the problem with Semantic Phenotypes and class-based TBox semantic graphs is the anonymity of the resources referenced in their class axioms. If you want to describe a given ODU using several Semantic Phenotypes, the entities, related entities, and qualities mentioned in the axioms of phenotype classes of different Semantic Phenotypes do not relate to each other, although they may actually refer to the same real entities, because they cannot be individually referenced and identified through the information provided by the graph. This is not the case with Phenotype Knowledge Graphs and instance-based ABox semantic graphs in general because each described part and property possesses its own URI and thus can be referred to in several different graphs.

#### Correcting mistakes in phenotype descriptions

Researchers are human beings, and human beings make mistakes. Therefore, phenotype descriptions should allow for effective ways to **correct for mistakes** and thereby unambiguously track what information has been changed and ideally document that change in RDF as well. And again, because Semantic Phenotypes cannot be easily fragmented and the particular parts, properties, and qualities referenced in class axioms do not possess their own URIs, explicitly tracking what information has been changed between the original Semantic Phenotype and the corrected version of that Semantic Phenotype, and documenting in RDF all the changes that have been made, is rather difficult to accomplish. Phenotype Knowledge Graphs, in contrast, can be easily corrected for mistakes. Because the descriptive statements of Phenotype Knowledge Graphs are organized into different named graphs, one can easily correct information in one of them and track provenance and relevant metadata for it, as well as document in the metadata all changes that have been made.

#### Universal usability and reusability of phenotype descriptions

Being able to fragment a Phenotype Knowledge Graph into smaller subgraphs allows using only those parts of the data that are relevant for a given research question, while ignoring all parts that are irrelevant. The differentiation of types of observation and the modelling of respective data into corresponding named graphs allows meaningful fragmentation of data and reuse in various frameworks. While this is in principle also possible with Semantic Phenotypes, the extraction of only the relevant data is not as straightforward.

#### Generation of phenotype descriptions

As already mentioned above, in order to generate a Semantic Phenotype, the corresponding phenotype must be first defined as an ontology class before the description itself can be generated, which in turn only specifies that a given ODU instantiates that specific class. Technically, the actual phenotype description is contained in the definition of the ontology class.

Defining such phenotype ontology classes is usually conducted using OWL Manchester Syntax, which can become very complex, especially if the underlying phenotype is complex and the description fine-grained. For instance the EQ statement *“head color: reddish brown, except for dark brown to black postgena, occiput, vertex; mandibles, maxillary and labial palps yellowish; scape, pedicel, F1 and F2 yellow, subsequent flagellomeres progressively darker”* translates to the OWL Manchester Syntax expression (example taken from suppl. Material 2 of [95]):

*has part* some ( head and ((not ( clypeus )) and (not ( mandible and ((((not ( antenna )) and ( *bearer of* some red )) and (not ( labial palp ))) and (not ( maxillary palp )))))) and ( *has part* some ( labial palp and ( *bearer of* some yellow ))) and ( *has part* some ( mandible and ( *bearer of* some yellow ))) and ( *has part* some ( maxillary palp and ( *bearer of* some yellow ))) and ( *has part* some ( occiput and ( *bearer of* some dark brown ))) and ( *has part* some ( pedicel and ( *bearer of* some yellow ))) and ( *has part* some ( postgena and ( *bearer of* some dark brown ))) and ( *has part* some ( scape and ( *bearer of* some yellow ))) and ( *has part* some ( vertex and ( *bearer of* some dark brown ))) and ( *has part* some ( first flagellomere and ( *bearer of* some yellow ))) and ( *has part* some ( second flagellomere and ( *bearer of* some yellow ))) and ( *has part* some ( fifth flagellomere and (( *bearer of* some color brightness ) and ( *increased in magnitude relative to* some ( color brightness and ( *inheres in* some sixth flagellomere )))) and ( *bearer of* some light brown ))) and ( *has part* some ( third flagellomere and (( *bearer of* some color brightness ) and ( *increased in magnitude relative to* some ( color brightness and ( *inheres in* some fourth flagellomere )))) and ( *bearer of* some light brown ))) and ( *has part* some ( fourth flagellomere and (( *bearer of* some color brightness ) and ( *increased in magnitude relative to* some ( color brightness and ( *inheres in* some fifth flagellomere )))) and ( *bearer of* some light brown ))) and ( *has part* some ( sixth flagellomere and (( *bearer of* some color brightness ) and ( *increased in magnitude relative to* some ( color brightness and ( *inheres in* some seventh flagellomere )))) and ( *bearer of* some light brown ))) and ( *has part* some ( seventh flagellomere and (( *bearer of* some color brightness ) and ( *increased in magnitude relative to* some ( color brightness and ( *inheres in* some eighth flagellomere )))) and ( *bearer of* some light brown ))) and ( *has part* some ( eighth flagellomere and (( *bearer of* some color brightness ) and ( *increased in magnitude relative to* some ( color brightness and ( *inheres in* some ninth flagellomere )))) and ( *bearer of* some light brown ))) and ( *has part* some ( ninth flagellomere and (( *bearer of* some color brightness ) and ( *increased in magnitude relative to* some ( color brightness and ( *inheres in* some tenth flagellomere )))) and ( *bearer of* some light brown ))) and ( *has part* some ( eleventh flagellomere and (( *bearer of* some color brightness ) and ( *increased in magnitude relative to* some ( color brightness and ( *inheres in* some twelfth flagellomere )))) and ( *bearer of* some light brown ))) and ( *has part* some ( twelfth flagellomere and (( *bearer of* some color brightness ) and ( *increased in magnitude relative to* some ( color brightness and ( *inheres in* some thirteenth flagellomere )))) and ( *bearer of* some light brown ))) and ( *has part* some ( thirteenth flagellomere and (( *bearer of* some color brightness ) and ( *increased in magnitude relative to* some ( color brightness and ( *inheres in* some flagellomere 14 )))) and ( *bearer of* some light brown )))).^15^

Obviously, respective class axioms can consist of many levels of nested expressions organized in parentheses, which many researchers have a hard time to read and comprehend. Also, this method of description is very error-prone due to this nested syntax. Alternatively, such OWL Manchester Syntax based expressions can be restricted to a certain threshold of slots. In Phenoscape, for example, templates are used with three slots. Restricting the descriptions to three slots keeps Semantic Phenotypes from getting too complicated, but also prevents them from being as precise and detailed as possible.

Another problem with the Semantic Phenotype approach becomes apparent when considering morphometric data.^16^ When describing phenotypes based on a set of multiple measurements, the Semantic Phenotype approach would require for every possible combination of measurements the definition of a corresponding phenotype class. With the addition of more quantitative properties, this would result in exponentially increasing numbers of possible phenotype classes. Documenting every type of measurement as a single Semantic Phenotype somewhat mitigates the problem but results in the above-mentioned problem of disconnected information due to anonymous resources.

Whereas the generation of Phenotype Knowledge Graphs does not face these problems, it requires the development of an adequate application that allows researchers describing phenotypes respectively. This application could utilize the hierarchical structure of parthood relations between described parts of a given description to organize its interface. For each description, the partonomy could be visualized as a tree-like structure of described parts. This partonomy could also function as a navigator for selecting a particular described part. Each part, in turn, has its own input form associated with it that allows a detailed description of that part and can be accessed by selecting the part within the partonomy. We are currently developing such an application for the online anatomical data repository Morph‧D‧Base [96] and a functional prototype is available. The interface has been developed in close cooperation with several anatomy-experts from different backgrounds, who served as use-cases during its development. They considerably contributed to it, allowing an intuitive generation of Phenotype Knowledge Graphs. All data is stored in a Jena tuple store and descriptions are organized into several description named graphs as described above. The interface provides a human-readable HTML-version of the description while retaining a machine-actionable and reasoning-capable version that can be accessed through a SPARQL endpoint, thus allowing exploiting semantic technology to its full potential and offering Phenotype Knowledge Graphs as Linked Open Data.

#### Potential suitability of ABox and TBox semantic graphs for data and metadata standards

In times of eScience, a standard for data and metadata must cover machine-actionability regarding terminological aspects relating to concepts (meaning) and nomenclature (reference) and assertional aspects relating to formats (syntax and file format) and contents (data model) [8, 9, 19, 31] (see Table 1). Moreover, it must also comply with the FAIR Guiding Principles [7, 97–99] (see Table 2).

**Table 1:** Potential suitability of TBox and ABox semantic graphs for meeting eScience-compliant data and metadata standards, using Semantic Phenotypes and Phenotype Knowledge Graphs as examples

**TERMINOLOGY**		
**Concept standard**		*What is the meaning of a concept? What do we know of the corresponding kind?*
Semantic Phenotype	✓✓	Reference to ontology terms provides machine- and human-readable specifications of the meaning of concepts used in data (i.e., phenotype descriptions) and metadata statements.
Phenotype Knowledge Graph	✓✓	
**Nomenclatural standard**		*Which words or symbols are used for referring to a specific kind?*
Semantic Phenotype	✓	URIs, preferred labels, and synonyms provide unambiguous reference of a kind term to its underlying class definition. However, entities (i.e., parts, properties, qualities, relations) mentioned in class axioms are referenced only anonymously.
Phenotype Knowledge Graph	✓✓	Same as with Semantic Phenotypes, with the addition that each particular descriptive statement, described part, property, quality, and relation of data and metadata statements of Phenotype Knowledge Graphs possess their own URI and can be individually referenced.
**ASSERTIONS**		
**Format standard**		*Which syntax and file format must be used?*
Semantic Phenotype	✓	Semantic Phenotypes can be documented in RDF/OWL, which provides a machine-actionable syntax and format. SPARQL can be used for querying, but querying is computationally more difficult than querying Phenotype Knowledge Graphs.
Phenotype Knowledge Graph	✓✓	Phenotype Knowledge Graphs can be documented in RDF/OWL, which provides a machine-actionable syntax and format. SPARQL can be used for querying. Querying Phenotype Knowledge Graphs is computationally less difficult than querying Semantic Phenotypes.
**Content standard**		*Which information is relevant? How must it be modeled?*
Semantic Phenotype	✓	The use of domain-specific semantic data models in Semantic Phenotypes provides a basic categorization and classification of contents relevant for a given domain.
Phenotype Knowledge Graph	✓✓	The use of domain-specific semantic data models in Phenotype Knowledge Graphs provides a basic categorization and classification of contents relevant for a given domain. With the identification of individual descriptive statements, parts, properties, qualities, and relations, Phenotype Knowledge Graphs can be categorized and classified at various levels of granularity, including levels finer than it is possible with Semantic Phenotypes.

**Table 2:** Potential suitability of TBox and ABox semantic graphs for meeting the FAIR Guiding Principles, using Semantic Phenotypes and Phenotype Knowledge Graphs as examples (criteria taken from [7], criteria for reusability not shown)

**FINDABLE**		
F1	*(meta) data are assigned a globally unique and persistent identifier*	
Semantic Phenotype	✓	Semantic Phenotypes reference ontology classes through their URIs, including the class defining the phenotype. Ontologies provide persistent identifiers for kind terms and their associated universal statements.
Phenotype Knowledge Graph	✓✓	Phenotype Knowledge Graphs not only reference ontology classes like Semantic Phenotypes do, but also provide URIs for every particular descriptive statement, described part, property, quality, and relation and thus for kind terms, universal statements, proper names, and assertional statements.
F2.	*data are described with rich metadata*	
Semantic Phenotype	✓	Metadata can be associated with a phenotype description as a whole, but not with each of the individual descriptive statements it comprises.
Phenotype Knowledge Graph	✓✓	Due to the possibility to organize a Phenotype Knowledge Graph into a set of named graphs, each of which documenting an individual descriptive statement, metadata can be associated on the fine granular level of particular descriptive statements of a phenotype description, in addition to the description as a whole.
F3.	*metadata clearly and explicitly include the identifier of the data it describes*	
Semantic Phenotype	✓	Metadata can include an identifier that refers to the description as a whole, but not to individual descriptive statements.
Phenotype Knowledge Graph	✓✓	Metadata can include an identifier that refers to the description as a whole, but also identifiers that refer to each individual descriptive statement.
F4.	*(meta) data are registered or indexed in a searchable resource*	
Semantic Phenotype	✓✓	Metadata can be expressed as TBox or ABox semantic graphs and stored in a tuple store.
Phenotype Knowledge Graph	✓✓	
**ACCESSIBLE**		
A1.	*(meta) data are retrievable by their identifier using a standardized communication protocol*	
Semantic Phenotype	✓	Semantic Phenotypes and their metadata can be stored in a tuple store and queried with SPARQL.
Phenotype Knowledge Graph	✓✓	Phenotype Knowledge Graphs and their metadata can be stored in a tuple store and queried with SPARQL. Because particular descriptive statements, described parts, properties, qualities, and relations have their own URIs, they can be individually accessed.
A1.1	*the protocol is open, free, and universally implementable*	
Semantic Phenotype	✓	SPARQL
Phenotype Knowledge Graph	✓	
A1.2	*the protocol allows for an authentication and authorization procedure, where necessary*	
Semantic Phenotype	–	This depends on the application employing the concept of Semantic Phenotypes or Phenotype Knowledge Graphs.
Phenotype Knowledge Graph	–	
A2.	*metadata are accessible, even when the data are no longer available*	
Semantic Phenotype	–	This depends on the application employing the concept of Semantic Phenotypes or Phenotype Knowledge Graphs.
Phenotype Knowledge Graph	–	
**INTEROPERABLE**		
I1.	*(meta) data use a formal, accessible, shared, and broadly applicable language for knowledge representation*	
Semantic Phenotype	✓	Semantic Phenotypes and Phenotype Knowledge Graphs both can be represented in RDF/OWL.
Phenotype Knowledge Graph	✓	
I2.	*(meta) data use vocabularies that follow FAIR principles*	
Semantic Phenotype	✓	Semantic Phenotypes and Phenotype Knowledge Graphs both use ontologies and other controlled vocabularies that provide URIs for their terms.
Phenotype Knowledge Graph	✓	
I3.	*(meta) data include qualified references to other (meta)data*	
Semantic Phenotype	✓	This depends on how and which (meta) data are provided, but Semantic Phenotypes and their associated metadata can include cross-references and inter-relationships to other Semantic Phenotypes and their metadata.
Phenotype Knowledge Graph	✓✓	This depends on how and which (meta) data are provided, but Phenotype Knowledge Graphs and their associated metadata can include cross-references and inter-relationships to other Phenotype Knowledge Graphs and their metadata and that to a finer degree of granularity than Semantic Phenotypes due to the fact that they provide URIs to individual descriptive statements and to each described part, property, quality, and relation.

An eScience-compliant **concept standard** requires a machine- and human-readable specification of the **meaning** of all concepts used in data and metadata statements. The specification provides information about what we know of the corresponding real universal, i.e., the kind. Semantic Phenotypes and Phenotype Knowledge Graphs both comply with this by referencing ontology terms that, in turn, provide unambiguous definitions of meanings for concepts both in human- and machine-readable ways.

The **nomenclatural standard** requires unambiguous specification of the **reference** of the words, symbols, and IDs used in data and metadata statements. It provides an unambiguous link between term and concept. Again, Semantic Phenotypes and Phenotype Knowledge Graphs both comply with this standard by using machine-readable persistent URIs in addition to human-readable labels for referring to ontology classes. The link between word, symbol, or ID and its corresponding concept, which in turn provides the meaning, is thus clear and unambiguous. This allows the reuse of ontology terms in any semantic graph without the necessity to include the entire ontology specification. However, only Phenotype Knowledge Graphs provide this standard also for all parts and properties mentioned in the description, which Semantic Phenotypes only reference anonymously.

The combination of concept and nomenclatural standard covers the **terminology-related** aspects of an eScience-compliant standard and ensures that phenotype descriptions are semantically transparent, allowing even non-experts to understand and interpret them correctly. In addition to these terminology-related aspects, eScience-compliant data and metadata standards must also cover assertions-related aspects, which is covered by a combination of a format and a content standard that ensures that phenotype descriptions are comparable, reusable, computer-parsable, and communicable through the Web.

The **format standard** requires a machine-readable specification of the syntax and file format to be used when documenting, storing, communicating, and processing data and metadata statements on the Web. Semantic Phenotypes and Phenotype Knowledge Graphs provide this through the possibility to store the respective semantic graphs in OWL files, which can be serialized to RDF. As a consequence, Semantic Phenotypes and Phenotype Knowledge Graphs both provide a basic level of findability, accessibility, and explorability because they can take the form of semantic graphs and any semantic graph can be searched using **SPARQL**. The query pattern of a SPARQL query is itself represented as a semantic graph that may contain variables and wildcards. The main mechanism of a SPARQL query is matching the query pattern with the semantic graph to be queried. A repository for Semantic Phenotypes or Phenotype Knowledge Graphs stored in a tuple store would allow searching for descriptions of heads of a specific taxonomic group that possess a specific type of antenna and that have a weight larger than 10 mg and retrieve a list of corresponding phenotype descriptions.

Regarding querying semantic graphs, however, it is important to note that querying TBox expressions is more difficult than querying ABox expressions. In case the graph contains class definitions in the form of axioms expressed in OWL, the basic graph-pattern-matching of SPARQL must be defined using entailment regimes [100]. Querying under entailment regimes is more complex and computationally difficult under full expressivity of OWL [101, 102]. As a consequence, querying Phenotype Knowledge Graphs is more straight forward and computationally less difficult than querying Semantic Phenotypes.

In ABox semantic graphs, we can associate a specific **content standard** for each descriptive named graph class. The content standard specifies the general structure of how to express the corresponding type of empirical information in terms of RDF triples by defining a corresponding **semantic graph pattern** [57], for example using an RDF graph schema language such as SHACL or ShEx. The same can be applied using TBox semantic graphs for standardizing the definitions of ontology classes. When applied consistently throughout a data repository that stores and manages phenotype descriptions, the set of templates would specify a **semantic model for phenotype data and metadata** [57]. Such data and metadata models would not only complement the format standard by further specifying the syntax of all types of descriptive and metadata statements relevant for phenotype descriptions but also specify the content standard aspect of eScience-compliant standards [8, 9, 19, 31]. The content standard requires the specification of **which information is relevant** for a specific type of data or metadata statement and provides a basic categorization and classification of possible contents belonging to a given domain and the corresponding schemata for modeling and documenting them. The graph pattern associated with each descriptive named graph class specifies which information must be provided for the given type of data statement. The same can be done with metadata statements, which should be associated with their own particular named graphs too. This significantly increases the comparability of Semantic Phenotypes and Phenotype Knowledge Graphs [57]*.*

Whereas the specification of templates for class axioms guarantee a certain level of comparability between different Semantic Phenotypes [51], these templates are very general and not customized to basic perceptual categories such as the semantic graph templates associated with Phenotype Knowledge Graphs. Therefore, Semantic Phenotypes are not to the same degree comparable with each other as Phenotype Knowledge Graphs.

Regarding the FAIR Guiding Principles, Phenotype Knowledge Graphs are slightly superior to Semantic Phenotypes with respect to the findability, accessibility, and interoperability criteria mentioned by Wilkinson et al. [7] (see Table 2). With respect to the criterion of reusability, the way phenotype data are represented is rather irrelevant, and it is more a question of implementation within an application and the quality of the metadata provided by the creators of a given phenotype description. Anyhow, what both representations of phenotypes lack is good human-readability of their data and associated metadata. This is a general problem with semantic graphs: whereas their machine-actionability can be excellent, their human-readability is usually poor—humans neither want to read RDF/OWL files nor triple statements or complex graphs. Moreover, since machines have problems with fuzzy and context-dependent information—something typically found in natural language texts—semantic graphs tend to be more complex and explicit than human readers need, adding information that human readers distract from the information they are interested in. Ideally, applications storing data in the form of semantic graphs feature tools that translate semantic graphs into human-readable statements that can be presented, for instance on an HTML page.

#### Machine-actionability of phenotype descriptions

As already discussed above, both Semantic Phenotypes and Phenotype Knowledge Graphs are machine-actionable. However, because each particular descriptive statement and described part, property, quality, and relation in a Phenotype Knowledge Graph possesses its own URI and reasoning over instance-based ABox semantic graphs is computationally less difficult than reasoning over class-based TBox semantic graphs, the machine-actionability of Phenotype Knowledge Graphs allows for broader practical applicability. Algorithms can use the information contained in a given set of Phenotype Knowledge Graphs together with the information contained in all ontologies they reference. By traversing the parthood hierarchy of a Phenotype Knowledge Graph and the class-subclass hierarchy of referenced ontologies, algorithms could match and map nodes between different Phenotype Knowledge Graphs and align them, in order to **identify units of comparison** between them and **measure the overall degree of similarity** between them [29]. Results of respective comparisons could themselves be documented as for instance separate consensus Phenotype Knowledge Graphs [29] that supplement the originally compared Phenotype Knowledge Graphs. The ability to measure the degree of similarity between a particular Phenotype Knowledge Graph and all Phenotype Knowledge Graphs stored in a phenotype repository would also greatly facilitate searching across phenotype descriptions, resulting in a search functionality comparable to the BLAST search for DNA sequences.

## Conclusion

Class-based and instance-based semantic representations of phenotypes are both overall FAIRer than phenotype descriptions in the form of unstructured natural language texts, especially regarding their machine-actionability. By linking URIs to corresponding ontology class definitions, they both provide unambiguous links to the meaning of the terms used in the descriptions and therewith provide the much-needed semantic transparency. This allows researchers to understand the descriptions, independent of their backgrounds within the life sciences and their expertise with the particular anatomy of the respective taxon. Moreover, when stored in adequate repositories, phenotype descriptions in the form of *S*emantic Phenotypes and Phenotype Knowledge Graphs become findable and accessible. Due to their use of URIs, searching a repository for specific phenotype data becomes possible. Searching for specific phenotype data in published literature, in contrast, is not only tedious and exhausting but significantly less efficient and often also hampered by pay-walls.

The incomprehensibility of phenotype descriptions for non-experts and their limited findability and accessibility has been one of the most detrimental problems of anatomy/morphology as a discipline in academia. If colleagues from other disciplines have problems finding your data and when they find them, they have problems understanding them, they will likely think twice to collaborate with you and are therefore less interested in your research. Both the Semantic Phenotype approach and the Phenotype Knowledge Graph approach have the potential to change this. Moreover, both approaches enable the application of machine-reasoning, which can be utilized for various analytical purposes, for inferencing, and for checking the consistency of the data [86, 103–105]. However, with respect to the FAIR Guiding Principles and their suitability for meeting eScience-compliant standards, the Phenotype Knowledge Graph approach is superior to Semantic Phenotypes, because querying its graphs is computationally less difficult and integrating metadata straight forward.

Apart from that, looking at the various practical implications of the technical differences between the two general approaches, the instance-based ABox semantic graphs approach seems to be in general superior to the class-based TBox semantic graphs approach in the context of documenting and managing empirical data in knowledge graphs, because it allows the identification of each particular descriptive statement, each described entity, quality, and relation, enabling the decomposition of the data graph into various fragments. This characteristic of instance-based ABox semantic graphs is **not limited to semantic descriptions of phenotypes**, but applies to the description of **any type of ODU**, including all sorts of particular material entities, spaces, and processes. Thus, when describing a particular entity, instance-based semantic graphs are in general superior to class-based semantic graphs for the same reasons that Phenotype Knowledge Graphs are superior to Semantic Phenotypes.

Coupled with their better querying properties, Phenotype Knowledge Graphs together with semantic technologies provide a promising framework for developing not only new innovative analytical methods but also new applications that will substantially support everyday research in the life sciences. We could, for instance, develop algorithms for taxonomists that facilitate **statistical evaluation** of species affiliation in an anatomically heterogeneous population based on phenotype descriptions. Once we can semi-automatically annotate images and automatically produce Phenotype Knowledge Graphs based on these annotations, the algorithms could compare these graphs and identify putative sub-populations and even suggest adequate diagnostic characters to differentiate these sub-populations. As soon as taxonomists then decide which specimen is the holotype, the algorithms could statistically evaluate the Phenotype Knowledge Graphs of all other previously described specimens belonging to the taxon and generate a consensus description containing all possible conditions found in that taxon. This could be done automatically, and the consensus description would be adjusted dynamically with every new specimen of that taxon being described. Phenotype Knowledge Graphs could even be used for taxonomically identifying the species affiliation of a described specimen. All the resulting information could be documented in a **Taxonomy Knowledge Graph**, which could provide various valuable services to the life science community, such as automatically generated dynamic multi-entry keys that could add annotated images to each of their decision points.

In any case, being able to represent the anatomy of particular specimens in a machine-readable and machine-actionable format is not only going to change the way anatomical research will be done in the future, but it will also increase the visibility and importance of anatomy/morphology and taxonomy as scientific disciplines. Exciting times ahead for morphologists!

## Data Availability

Not applicable.

## Abbreviations

DL: Description logics
EAV: Entity-Attribute-Value
EQ statement: Entity Quality statement for representing characters
E (QC): Entity Quality statement with a count element for representing quantitative characters
E (QRE): Entity Quality statement with inclusion of a related entity for representing relational characters
FAIR: Findable, Accessible, Interoperable, Reusable
HTML: Hypertext Markup Language
ODU: Operational Descriptive Unit
OWL: Web Ontology Language
RDF: Resource Description Framework
SHACL: Shapes Constraint Language
ShEx: Shape Expressions
SPARQL: SPARQL Protocol And RDF Query Language
URI: Uniform Resource Identifier

## Footnotes

1 The Semantic Web is an extension of the World Wide Web based on standards provided by the World Wide Web Consortium (W3C) that promote common data formats and exchange protocols. The aim of the Semantic Web is to enable data to be easily shared and reused through the Web by making them machine-actionable.

2 OWL is a Semantic Web language. It is used to represent knowledge about particular entities or kinds of entities, their properties, and relations between them in a computational, logic-based syntax and format that is machine-actionable. Computer programs can verify the consistency of knowledge contained in an OWL file or deduce implicit knowledge from it. Many ontologies are stored as OWL files.

3 RDF is a standardized model for interchanging data on the Web. OWL files can be serialized to RDF—RDF is the primary exchange syntax for OWL.

4 OWL distinguishes three types of resources: classes, instances, and properties. Classes are instantiated by instances. All instances of a class usually share one or more characteristics and represent the extension of the class. Instances are related to one another, and to literal values, via OWL properties. OWL classes are described through class descriptions. Class descriptions include, among others, specification of the class identifier (i.e., its URI), intersection of two or more class descriptions, union of two or more class descriptions, complement of a class description, and property restrictions. When combined, several class descriptions form class axioms.

5 Ontology classes are described using axioms, i.e., propositions that define the class in a logical form, specifying the necessary and sufficient conditions for an individual entity to be an instance of that class.

6 A universal statement is a proposition that states that all entities of a specific kind have a particular property. A proposition stating that a given individual entity is an instance of a specific kind of entity that has specific properties, on the other hand, represents a particular empirical observation.

7 A URI unambiguously identifies a particular Web resource, i.e., any identifiable digital, physical, or abstract thing. Uniformity of URIs is guaranteed through a predefined set of syntax rules. The most common form of URI is the Uniform Resource Locator (URL).

8 Throughout the paper, we use regular underlined for representing ontology classes with their labels and *italic underlined* for representing ontology properties with their labels.

9 Essential properties of a specific kind of entity refer to the properties that all instances of the kind *must* have.

10 Analogue to the Operational Taxonomic Unit (OTU), which refers to some taxonomic entity, the ODU refers to the object being described. It is the referent of the description and, in the case of anatomical phenotype descriptions, refers to some anatomical entity.

11 One might argue that for morphologists/taxonomists, phenotype descriptions are only ‘figure captions’ (pers. comm. István Mikó) and images are more important than phenotype descriptions. However, images stored in a database without the corresponding captions and without structured annotations are increasingly difficult to find with an increasing number of images in the database, turning the database eventually into an image cemetery. Also, for analytical purposes, it is usually necessary to translate the perceptual contents of images into textual contents [31]. Logical inferences and tests against consistency can only be performed against textual contents.

12 SPARQL is a query language for RDF [84].

13 This, however, could also be accomplished using an RDF graph schema language such as SHACL or ShEx.

14 *has component* is used instead of *has part* because the latter is a transitive object property and OWL does not permit cardinality constraints to be used in combination with transitive object properties.

15 Ontology classes shown with their label in regular underlined, ontology properties with their label in *italics and underlined*, ‘and’ being used in the sense of intersection of two sets and ‘some’ in the sense of the existential quantifier ‘there exists a’ or ‘some instance of’.

16 Quantitative data about the size, shape, and spatial position of anatomical entities.
